# Transcutaneous Application of Carbon Dioxide (CO_2_) Induces Mitochondrial Apoptosis in Human Malignant Fibrous Histiocytoma *In Vivo*

**DOI:** 10.1371/journal.pone.0049189

**Published:** 2012-11-15

**Authors:** Yasuo Onishi, Teruya Kawamoto, Takeshi Ueha, Kenta Kishimoto, Hitomi Hara, Naomasa Fukase, Mitsunori Toda, Risa Harada, Masaya Minoda, Yoshitada Sakai, Masahiko Miwa, Masahiro Kurosaka, Toshihiro Akisue

**Affiliations:** 1 Department of Orthopaedic Surgery, Kobe University Graduate School of Medicine, Kusunoki-cho, Chuo-ku, Kobe, Japan; 2 NeoChemir Inc., Gokodori, Chuo-ku, Kobe, Hyogo, Japan; 3 Faculty of Health Care Sciences, Himeji Dokkyo University, Kami-Ohno, Himeji, Hyogo, Japan; University Hospital of Modena and Reggio Emilia, Italy

## Abstract

Mitochondria play an essential role in cellular energy metabolism and apoptosis. Previous studies have demonstrated that decreased mitochondrial biogenesis is associated with cancer progression. In mitochondrial biogenesis, peroxisome proliferator-activated receptor gamma coactivator-1 alpha (PGC-1α) regulates the activities of multiple nuclear receptors and transcription factors involved in mitochondrial proliferation. Previously, we showed that overexpression of PGC-1α leads to mitochondrial proliferation and induces apoptosis in human malignant fibrous histiocytoma (MFH) cells *in vitro*. We also demonstrated that transcutaneous application of carbon dioxide (CO_2_) to rat skeletal muscle induces PGC-1α expression and causes an increase in mitochondrial proliferation. In this study, we utilized a murine model of human MFH to determine the effect of transcutaneous CO_2_ exposure on PGC-1α expression, mitochondrial proliferation and cellular apoptosis. PGC-1α expression was evaluated by quantitative real-time PCR, while mitochondrial proliferation was assessed by immunofluorescence staining and the relative copy number of mitochondrial DNA (mtDNA) was assessed by real-time PCR. Immunofluorescence staining and DNA fragmentation assays were used to examine mitochondrial apoptosis. We also evaluated the expression of mitochondrial apoptosis related proteins, such as caspases, cytochorome c and Bax, by immunoblot analysis. We show that transcutaneous application of CO_2_ induces PGC-1α expression, and increases mitochondrial proliferation and apoptosis of tumor cells, significantly reducing tumor volume. Proteins involved in the mitochondrial apoptotic cascade, including caspase 3 and caspase 9, were elevated in CO_2_ treated tumors compared to control. We also observed an enrichment of cytochrome c in the cytoplasmic fraction and Bax protein in the mitochondrial fraction of CO_2_ treated tumors, highlighting the involvement of mitochondria in apoptosis. These data indicate that transcutaneous application of CO_2_ may represent a novel therapeutic tool in the treatment of human MFH.

## Introduction

Musculoskeletal malignancies, particularly high-grade sarcomas such as malignant fibrous histiocytoma (MFH), are clinically aggressive and demonstrate high metastatic behavior in various organs. Although many chemotherapeutic protocols are used to treat human sarcomas, current treatment strategies for high-grade sarcomas are ineffective and the prognosis of patients is poor due to local recurrence and metastases [1]. Therefore, new therapeutic strategies against high-grade sarcomas are required.

Mitochondria are cytoplasmic organelles that play an essential role in cellular energy metabolism and programmed cell death [2]. Previous studies have linked decreases in mitochondrial metabolism and/or mitochondrial number to cancer progression [3], [4], [5]. Mitochondrial proliferation has also been shown to play an important role in cellular apoptosis and may be an integral part of a cascade of apoptotic events [6]. Peroxisome proliferator-activated receptor gamma coactivator-1 alpha (PGC-1α) is a multi-functional transcriptional coactivator that regulates the activities of multiple nuclear receptors and transcription factors involved in mitochondrial biogenesis [7]. Specifically, PGC-1α transcriptionally regulates the gene encoding mitochondrial transcription factor A (TFAM), which plays an important role in mitochondrial biogenesis [8]. TFAM expression mirrors the fluctuating levels of mitochondrial DNA (mtDNA) in the cell, and mitochondrial synthesis is stimulated by the PGC-1α/TFAM pathway [8]. We have previously shown that mitochondria abundance is significantly decreased in several human sarcomas compared to benign tumors (unpublished data). Furthermore, we demonstrated that PGC-1α overexpression increases mitochondrial proliferation and induces mitochondrial apoptosis in human MFH cells *in vitro* (unpublished data). These results suggest that regulation of mitochondrial proliferation via modulation of PGC-1α expression, may be utilized as a useful therapeutic tool for the treatment of human musculoskeletal malignancies.

Carbon dioxide (CO_2_) therapy in the form of a carbonated spa has been historically used in Europe as an effective treatment for cardiac diseases and skin lesions [9], [10]. The therapeutic effects of CO_2_ are caused by an increase in blood flow and microcirculation, nitric oxide-dependent neocapillary formation, and a partial increase in O_2_ pressure in the local tissue, known as the Bohr effect [9], [10], [11]. Previously, we demonstrated that our transcutaneous CO_2_ therapy to rat skeletal muscle induced PGC-1α expression, and led to an increase in mitochondria [12]. These findings suggest that our transcutaneous CO_2_ therapy can upregulate the mitochondrial biogenesis through an increase of PGC-1α expression in the treated tissue.

Based on our previous studies in skeletal muscle, we hypothesized that transcutaneous application of CO_2_ may also induce PGC-1α expression and mitochondrial proliferation in tumor tissue, but in this context lead to tumor cell apoptosis. In this study, we use a murine model of human MFH to investigate the effects of transcutaneous application of CO_2_ on mitochondrial biogenesis and tumor cell apoptosis.

## Results

### Transcutaneous Application of CO_2_ Significantly Reduced MFH Cell Growth *in vivo*

**Figure 1:**
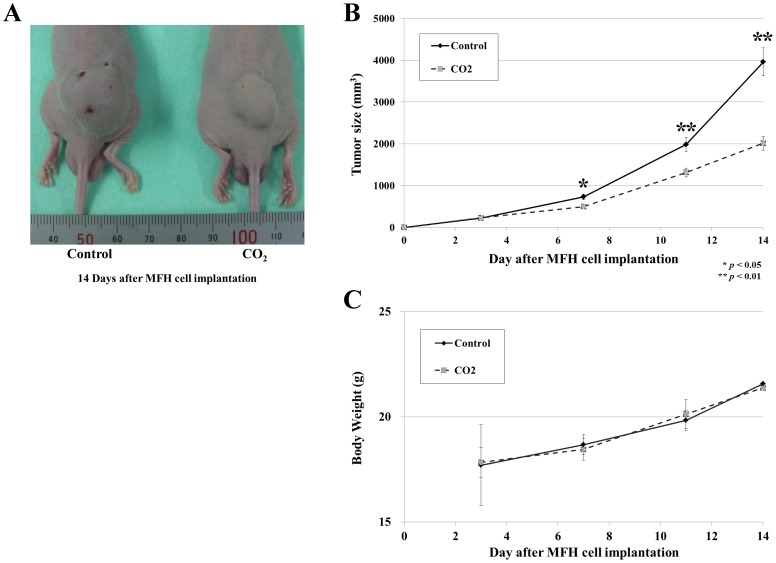
Effect of transcutaneous application of CO_2_ on MFH cell growth *in vivo*. Mice were treated with CO_2_ or control air three days after MFH cell implantation. Treatment was administered twice weekly for two weeks. (A) MFH tumors in CO_2_ treated and control mice, two weeks post-implantation. (B) Tumor volume (mm^3^) in CO_2_ treated or control mice was monitored for two weeks post-implantation. (C) Body weight (g) of CO_2_ treated or control mice was monitored for two weeks post-implantation. Data represent the mean ± S.E of at least three independent experiments (*p<0.05, **p<0.01).

To determine the effect of our CO_2_ treatment on MFH cell growth *in vivo*, we constructed a murine model of human MFH by transplanting the Nara-H cell line into the dorsal subcutaneous area of mice. Transcutaneous application of CO_2_ reduced tumor volume by 48% in treated mice compared to controls (p<0.01) (Figure 1A and B). No significant difference in body weight was observed between CO_2_ treated and control groups (Figure 1C). Thus, transcutaneous application of CO_2_ had an inhibitory effect on MFH tumor growth *in vivo*, with no observable negative side effects.

### Transcutaneous Application of CO_2_ Up-regulated the PGC-1α-TFAM-mitochondria pathway

**Figure 2:**
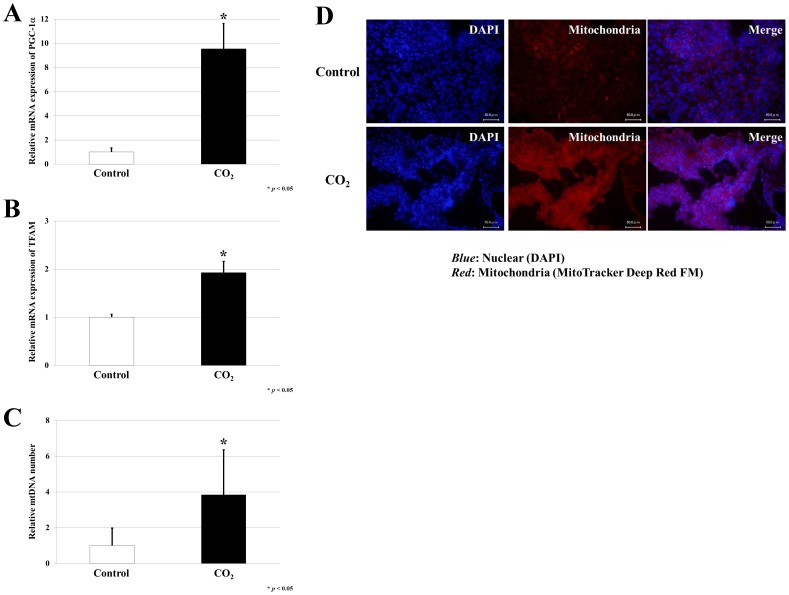
Effect of transcutaneous application of CO_2_ treatment on mitochondrial proliferation in tumors. qRT-PCR for *PGC-1α* (A) and *TFAM* (B) in CO_2_ treated or control tumor specimens collected two weeks post-treatment. Expression was normalized to *β-actin* control. Data represent the mean ± S.E of at least three independent experiments (*p<0.05). (C) mtDNA was measured in CO_2_ treated or control tumor samples by PCR and the relative copy number was determined by normalizing to nDNA. Data represent the mean ± S.E. of at least three independent experiments (*p<0.05). (D) Immunofluorescence staining of mitochondria in CO_2_ treated or control tumors after two weeks (*Blue*, nuclear; *Red*, mitochondria).

To investigate the mechanisms underlying the decrease in tumor volume in CO_2_ treated mice, we examined the expression of PGC-1α and TFAM in tumor tissue using quantitative real-time PCR (qRT-PCR). PGC-1α and TFAM expression was significantly increased in the CO_2_ group compared to control animals (p<0.05) (Figure 2A and B). Previous studies have shown that mitochondrial synthesis is stimulated by the PGC-1α/TFAM pathway [8]. Thus, we measured the relative levels of mtDNA to nuclear DNA (nDNA) in both CO_2_ treated and control groups. mtDNA copy number was significantly higher in the tumors from CO_2-_treated animals compared to controls (p<0.05) (Figure 2C). Consistent with these findings, immunofluorescence staining of mitochondria revealed that mitochondria levels were elevated in the CO_2_ treated tumors relative to controls (Figure 2D). Staining of normal muscle tissues as a positive control revealed strong staining (Figure S1).

### Mitochondrial Apoptosis was Induced by Transcutaneous Application of CO_2_ Treatment in Human MFH Cells *in vivo*

**Figure 3:**
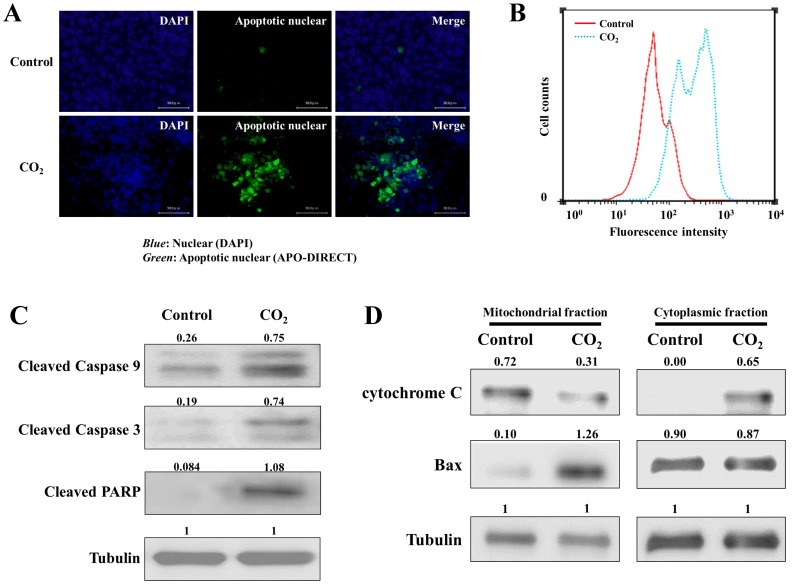
Evaluation of mitochondrial induced apoptosis in CO_2_ or control treated tumors. (A) DNA fragmentation analysis of tumor samples from CO_2_ treated and control mice two weeks post-treatment by immunofluorescence. (*Blue*, nuclear; *Green*, apoptosis nuclear) (B) DNA fragmentation was assessed by flow cytometry in CO_2_ treated tumors (*Blue dots*) and control tumors (*Red*) two weeks post-treatment. (C) Immunoblot analyses determined that increased expression of the cleavage products of caspase 3 and 9, and PARP occurred in the CO_2_ treated tumors compared to the control tumors. Tubulin was used as an endogenous loading control. (D) Immunoblot analysis of cytochrome c and Bax in mitochondrial and cytoplasmic fractions of CO_2_ treated and control tumors. Tubulin was used as an endogenous loading control. (C, D) Positive bands in immunoblot analyses were semiquantified using densitometrical analyses using the Image J program (NIH, USA, http://rsb.info.nih.gov/ij/).

We performed immunofluorescence staining for DNA breaks to evaluate the effect of CO_2_ treatment on MFH cell apoptosis *in vivo*. We observed an increase in cells with apoptotic nuclei in tumors from the CO_2_ treated group compared to controls (Figure 3A). Flow cytometry revealed that DNA fragmentation, a measure of apoptosis, was increased in CO_2_ treated tumors compared to controls (Figure 3B). Taken together, these results indicate that CO_2_ treatment induced apoptosis in human MFH cells *in vivo*.

We also examined the cleavage of caspases and PARP, and evaluated the expression of cytochrome c and Bax in the mitochondrial and cytoplasmic fractions separately to determine the involvement of mitochondria in the observed apoptosis. Immunoblot analyses revealed increased cleavage products of caspase 3 and 9, and PARP in CO_2_ treated tumors, but not in the control tumors (Figure 3C). Furthermore, we observed decreased expression of cytochrome c in the mitochondrial fraction and increased expression in the cytoplasmic fraction in the CO_2_ treated group compared to controls. Conversely, Bax protein was increased in the mitochondrial fraction and was decreased in the cytoplasmic fraction (Figure 3D). Positive bands in immunoblot analyses were semiquantified using densitometrical analyses using the Image J program (NIH, USA, http://rsb.info.nih.gov/ij/). Taken together, these results indicated that the anti-tumoral effect of transcutaneous CO_2_ treatment in a murine model of human MFH may be mediated via mitochondria induced apoptosis.

### Transcutaneous Application of CO_2_ Treatment Increased Intracellular Ca^2+^ in MFH Cells

**Figure 4:**
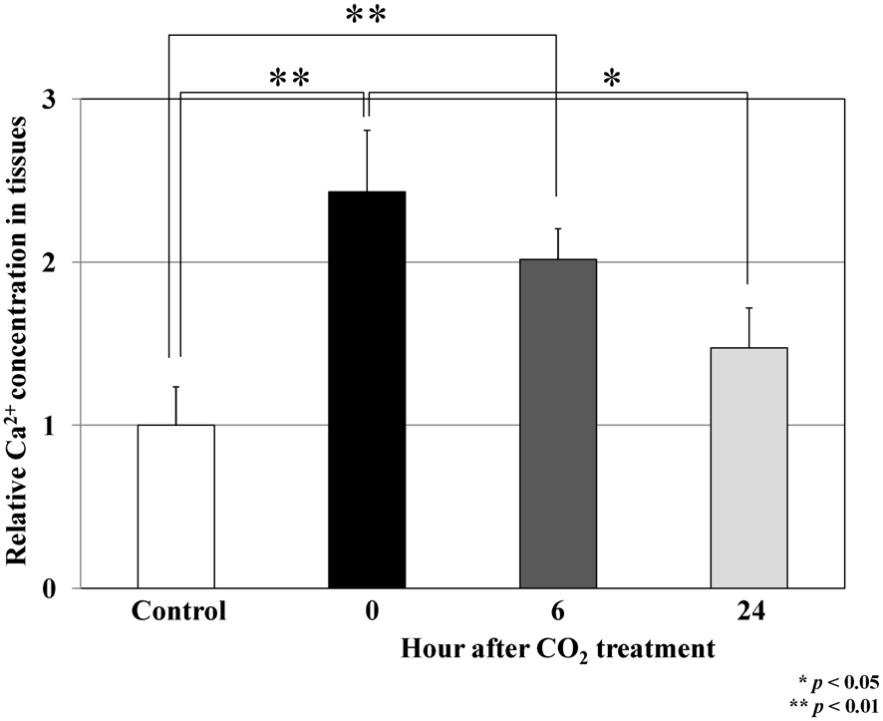
Effect of transcutaneous CO_2_ application on intracellular Ca^2+^ concentration in a mouse model of human MFH. Implanted tumors were isolated from mice at 0 (n = 12), 6 (n = 6) and 24 hours (n = 12) after transcutaneous CO_2_ exposure, and the intracellular Ca^2+^ concentration was assessed using the Calcium Assay Kit. Data represent the mean ± S.E. of at least three independent experiments (*p<0.05, **p<0.01).

We finally investigated the mechanism of the induction of the PGC-1α-TFAM-mitochondria pathway by our system in MFH cells. It has been reported that raising intracellular calcium (Ca^2+^) concentration induces the PGC-1α expression [13], 14, and mitochondrial biogenesis [13], [14]. Therefore, we examined the effect of our CO_2_ treatment on the intracellular Ca^2+^ concentration in human MFH cells *in vivo*. We isolated implanted tumors from mice at 0, 6 and 24 hours after our transcutaneous CO_2_ treatment, and we evaluated the intracellular Ca^2+^ in the tumors. At 0 and 6 hours after treatment, Ca^2+^ concentration in CO_2_ treated tumors was significantly higher than that in the control tumors (Figure 4). The elevated relative Ca^2+^ concentration of the CO_2_ treated cells fell in a time-dependent manner after treatment and was equivalent to that of the cells in untreated control tumors within 24 hours (Figure 4). The results indicated that transcutaneous CO_2_ exposure increased the intracellular Ca^2+^ concentration in human MFH *in vivo*.

## Discussion

A number of studies have shown that decreased mtDNA levels are associated with neoplastic transformation and/or tumor progression [15], [16], and that mtDNA plays an important role in apoptosis [17]. Higuchi *et al*. investigated the role of mitochondrial respiration in apoptosis signaling in the human myelogenous leukemia cell line, ML-1a. These studies revealed that respiration-deficient clones were resistant to the tumor necrosis factor-induced apoptosis, whereas the clones reconstituted with normal mtDNA were sensitive [18]. We previously demonstrated that both mtDNA levels and PGC-1α expression were significantly decreased in human sarcoma tissues (unpublished data). Our studies showed that apoptosis could be induced by PGC-1α overexpression, which led to an increase in mtDNA number in human MFH cells (unpublished data). These results indicated that loss of PGC-1α expression and the subsequent decrease in mtDNA may render cells resistant to a certain apoptotic pathway.

The relationship between cancer and CO_2_ is controversial [19], [20], [21]. It is well established that CO_2_ alters the functions of macrophages and polymorphonuclear cells in the peritoneal and thoracic cavity [22]. While studies exposing tumor cells to CO_2_ are not conclusive [23], numerous studies confirm that CO_2_ affects the behavior of tumor cells derived from colon carcinomas, adenocarcinomas and breast cancers [19], [20], [24]. Although several studies have identified a CO_2_-associated increase in tumor cell growth and invasiveness in various cancer cell lines [19], [21], there are also reports that show CO_2_ can inhibit tumor cells [23], [24]. Gutt *et al*. demonstrated that CO_2_ increased cell necrosis and decreased proliferation in colonic and pancreatic carcinoma cells [25]. Hao *et al*. reported that exposure of gastric cancer cells to CO_2_ pneumoperitoneum significantly induced apoptosis [26]. In the current study, we demonstrate that transcutaneous application of CO_2_ to human MFH cells in an engrafted tumor model, upregulated the expression of PGC-1α and TFAM, increased the number of mitochondria, and led to mitochondrial-induced apoptosis. Furthermore, we previously observed a similar effect on human breast cancer cells *in vivo* (Figure S2). Therefore, our transcutaneous CO_2_ therapy may have an antitumoral effect on various human malignancies. However, the mechanisms underlying this observation remain unknown. In muscle tissue, mitochondrial respiration is regulated by PGC-1α, which stimulates various genes associated with mtDNA replication and transcription [7]. Generally, PGC-1α is induced by exercise in muscles, and mediates known responses to exercise such as muscle fiber-type switching and mitochondrial biogenesis [27]. PGC-1α expression is also induced by other stimuli, such as thyroid hormone treatment or 5-aminoimidazole-4-carboxamide-1-β-d-ribofuranoside (AICAR)-induced AMPK activation [28], as well as contractile activity in skeletal muscle [28], [29]. Several signaling kinases, after activation of calcium influx, such as p38 [30], AMPK [31] and CaMKIV [32], have also been implicated in mediating transcriptional activation of PGC-1α [33]. We recently demonstrated that transcutaneous application of CO_2_ upregulates PGC-1α expression in rat skeletal muscle, establishing a potential link between CO_2_ exposure and the induction of mitochondrial biogenesis [12]. It is reported that CO_2_ increased the intracellular Ca^2+^ concentration in various cells [34], [35], and that the increase in intracellular Ca^2+^ increases the expression of PGC-1α and the amount of mitochondria [13], [14], [36]. These reports indicated that CO_2_ induced the PGC-1α expression and mitochondrial biogenesis through raising the intracellular Ca^2+^ concentration. In the current study, we have demonstrated that our transcutaneous CO_2_ treatment increased the intracellular Ca^2+^ in human MFH cells *in vivo*. The results strongly support that the effect of our transcutaneous CO_2_ system on the induction of mitochondrial apoptosis through PGC-1α expression was caused by raising intracellular Ca^2+^ concentration.

We here show that localized, transcutaneous application of CO_2_ to an *in vivo* model of human MFH led to mitochondria-mediated apoptosis and impaired tumor growth, with no observable effects on body weight, a side effect typically observed following chemotherapy. Although further studies are needed to elucidate the mechanisms of the effects of the treatment on tumor cell apoptosis, our data indicate that transcutaneous application of CO_2_ may be a useful therapeutic tool for human MFH.

## Materials and Methods

### Cell Culture

The human MFH cell line, Nara-H (ScienStuff Co., Nara, Japan) [37], was used in this study. Cells were grown in Dulbecco’s Modified Eagle’s Medium (Sigma-Aldrich Co., St Louis, MO, USA) supplemented with 10% (v/v) fetal bovine serum (Sigma-Aldrich) and 100 U/ml penicillin/streptomycin solution (Sigma-Aldrich). Cells were maintained at 37°C in a humidified 5% CO_2_ atmosphere.

### Animal Models

Male athymic BALB/c nude mice, aged 5–8 weeks were obtained from CLEA Japan, Inc (Tokyo, Japan). Animals were maintained under pathogen-free conditions, in accordance with institutional principles. All animal experiments were performed according to the Guide for the Care and Use of Laboratory Animals at the host institution and were approved by the institutional animal committee (P-101203). Nara-H cells (4.0×10^6^ cells in 500 µl PBS) were injected into dorsal, subcutaneous area of mice as previously described [38].

### Transcutaneous CO_2_ Treatment

Transcutaneous application of CO_2_ was performed as previously described [12]. Briefly, the area of skin around the implanted tumor was treated with CO_2_ hydrogel. This area was then sealed with a polyethylene bag and 100% CO_2_ gas was administered into the bag (Figure S3). Each treatment was performed for 10 minutes. Control animals were treated similarly, replacing CO_2_ with an ambient air.

### *In vivo* MFH Tumor Studies

Twenty-four mice were randomly divided into two groups: CO_2_ group (n = 12) and control group (n = 12). Treatment commenced three days after MFH cell implantation, and was performed twice weekly for 2 weeks. Tumor volume and body weight in mice were monitored twice weekly until the end of the treatment. Tumor volume was calculated as previously described [38] according to the formula V = π/6×a^2^×b, where a and b represent the shorter and the longer dimensions of the tumor, respectively. At the completion of treatment, all tumors were excised from mice and tissue was stored at −80°C.

### Quantitative Real-time PCR

The mRNA expression of PGC-1α and TFAM in implanted tumors was analyzed by quantitative real-time PCR (qRT-PCR) [39]. Total RNA was extracted from tumor tissues by selective binding to a silica-gel-based membrane using an RNeasy Mini Kit, following the manufacturer’s protocol (QIAGEN, Valencia, CA, USA). cDNA was reverse transcribed with 1 µg of total RNA and oligo dT primer by MuLV reverse transcriptase (Applied Biosystems, Foster City, CA, USA). qRT-PCR was performed in a 20 µl reaction using SYBR Green Master Mix reagent (Applied Biosystems) on the ABI prism 7500 sequence detection system (Applied Biosystems). PCR conditions were as follows: 1 cycle at 95°C for 10 minutes followed by 40 cycles at 95°C for 15 seconds and 60°C for 1 minute. Pre-designed primers specific for human *PGC-1α*, human *TFAM* and human *β-actin* were obtained from Invitrogen (Carlsbad, CA, USA). Primer sequences were: PPARGC1A (that encodes PGC-1α), 5′-GGCAGAAGGCAATTGAAGAG-3′ (forward) and 5′-TCAAAACGGTCCCTCAGTTC-3′ (reverse); TFAM, 5′-CCGAGGTGGTTTTCATCTGT-3′ (forward) and 5′-GCATCTGGGTTCTGAGCTTT-3′ (reverse); β-actin, 5′-GATCATTGCTCCTCCTGAGC-3′ (forward) and 5′-ACATCTGCTGGAAGGTGGAC-3′ (reverse). The relative expression of PGC-1α and TFAM was calculated using the delta-delta Ct method, normalizing to β-actin.

### Evaluation of Mitochondrial Proliferation

Mitochondrial proliferation was assessed by determining the relative amount of mtDNA to nuclear (nDNA) in tumor samples. Genomic DNA was isolated from tumor specimens using the GenElute Mammalian Genomic DNA Miniprep Kit (Sigma-Aldrich), and PCR was performed using SYBR Green PCR Master Mix (Applied Biosystems) with primers designed to amplify a region corresponding to nucleotides 16–408 of a D-loop of human mtDNA. The primers used were 5′-GCAGATTTGGGTACCACCCAAGTATTGACTCACCC-3′ (forward) and 5′-GCATGGAGAGCTCCCGTGAGTGGTTAATAGGGTGATAG-3′ (reverse).

### Immunofluorescence Staining

To assess the mitochondrial proliferation and the apoptotic activity in treated tumors, we performed the immunofluorescence staining using the MitoTracker Deep Red FM (Invitrogen) and the APO-DIRECT Kit (BD Pharmingen, Franklin Lakes, NJ, USA) following the manufacturer’s protocol, respectively. The nucleus was stained with DAPI. The images were obtained using a BZ-8000 confocal microscope (Keyence).

### DNA Fragmentation Analysis

DNA fragmentation was evaluated using the APO-DIRECT Kit according to the manufacturer’s protocol (BD Pharmingen). Briefly, implanted tumors were excised, minced and filtered through a cell strainer (BD Falcon, Bedford, MA, USA) to obtain a single cell suspension. Erythrocytes were lysed in BD Pharm Lyse™ Lysing Buffer (BD Pharmingen) and the remaining cells were pelleted and resuspended in PBS. Single cell suspensions were fixed with 1% (v/v) paraformaldehyde and resuspended in 70% (v/v) ice cold ethanol at a concentration of 1×10^6^ cells/ml. Each cell pellet was resuspended in 50 µl of DNA Labeling Solution (Reaction Buffer: 10 µl, TdT Enzyme: 0.75 µl, FITC dUTP: 8.0 µl, distilled H_2_O: 32.25 µl) and incubated for 60 minutes at 37°C. FITC-dUTP-labeled cells were analyzed by the flow cytometry with a 520 nm Argon laser.

### Immunoblot Analysis

Cell lysates were prepared from tumor tissues using a whole cell lysis buffer (Mammalian Protein Extraction Reagent; Thermo Scientific, Rockford, IL, USA) supplemented with protease and phosphatase inhibitors (Roche Applied Science, Indianapolis, IN, USA). Mitochondrial fractions and cytoplasmic fractions were isolated using the Mitochondria Isolation Kit according to manufacturer’s protocol (Thermo Scientific), Protein concentration was quantified using the Bradford Protein Assay reagent (Bio-Rad, Richmond, CA, USA) and samples were processed using standard western immunoblotting procedures [39]. Membranes were incubated overnight at 4°C with the following antibodies in Can Get Signal Solution 1 (TOYOBO Co., LTD, Osaka, Japan): anti-human cleaved caspase 3 antibody (1∶1000) (Cell Signaling Technology, Danvers, MA, USA), anti-human cleaved caspase 9 antibody (1∶1000) (Cell Signaling Technology), anti-human cleaved PARP antibody (1∶1000) (Cell Signaling Technology), anti-human cytochrome c antibody (1∶1000) (eBiosience Inc., San Diego, CA, USA), anti-human Bax antibody (1∶1000) (Cell Signaling Technology) and anti-human α-tubulin antibody (1∶2000) (Sigma-Aldrich). Following washes, membranes were incubated with the appropriate secondary antibody conjugated to horseradish peroxidase, and exposed with ECL Plus Western blotting detection system reagent (GE Healthcare Bio-Sciences, Piscataway, NJ, USA). The signals were detected using the Chemilumino analyzer LAS-3000 mini (Fujifilm, Tokyo, Japan).

### Measurement of Intracellular Ca^2+^

To investigate the effect of transcutaneous CO_2_ treatment on intracellular Ca^2+^ in MFH tumor tissues, we isolated implanted tumors from mice at 0 (n = 12), 6 (n = 6) and 24 hours (n = 12) after our transcutaneous CO_2_ treatment, and evaluated the intracellular Ca^2+^ concentration using Calcium Assay Kit according to the manufacturer’s protocol (Cayman Chemical Company, Ann Arbor, Michigan, USA). Briefly, implanted tumors were excised, minced and rinsed with PBS containing 0.16 mg/ml heparin to remove any extraneous red blood cells and clots, and the tissues were homogenized in PBS containing 0.16 mg/ml heparin. Suspensions were centrifuged at 10000×g for 15 minutes at 4°C, and the supernatant was removed. Then, the detector was added, and the optical density was measured at a wavelength of 570 nm using a Model 680 Microplate Reader (Bio-Rad) after 5 minutes of incubation. The relative number of Ca^2+^ concentration was calculated.

### Statistical Analyses

Experiments were performed independently at least three times, and data are presented as the mean ± standard error unless otherwise indicated. Significance of differences between groups was evaluated using a two-tailed Student’s *t*-test, and by ANOVA with post hoc test to compare for continuous values. All tests were considered significant at *p*<0.05.

## Supporting Information

Figure S1 Immunofluorescence staining were performed in normal muscle tissues of mice as the control images of staining using the MitoTracker Deep Red FM (Invitrogen). The nucleus was stained with DAPI. The images were obtained using a BZ-8000 confocal microscope (Keyence).
(TIFF)

Figure S2 Effect of our transcutaneous CO_2_ treatment on the in vivo tumor growth of human breast cancer cell line, MDA-MB-231. Tumor model mice were created by subcutaneous implantation of the cells (1.5×10^6^ cells in 500 µl PBS). Mice were randomly divided into CO_2_ group (n = 5) or control group (n = 5), and treatment was performed twice weekly for 15 days. Tumor volume (A) and body weight (B) in mice were monitored until the end of the treatment. (A) At the end of the treatment, we observed a significant decrease in tumor volume in CO_2_ group compared with the control group (*p<0.05). (B) No significant difference in body weight was observed between CO_2_ treated and control groups.
(TIFF)

Figure S3 Transcutaneous application of CO_2_ for a model mouse of human MFH.
(TIFF)
