# Supplementary figures and images for: Transcutaneous Application of Carbon Dioxide (CO2) Induces Mitochondrial Apoptosis in Human Malignant Fibrous Histiocytoma In Vivo
Source: PLoS One. 2012 Nov 15;7(11):e49189. doi: 10.1371/journal.pone.0049189 (PMC3499556; doi:10.1371/journal.pone.0049189)

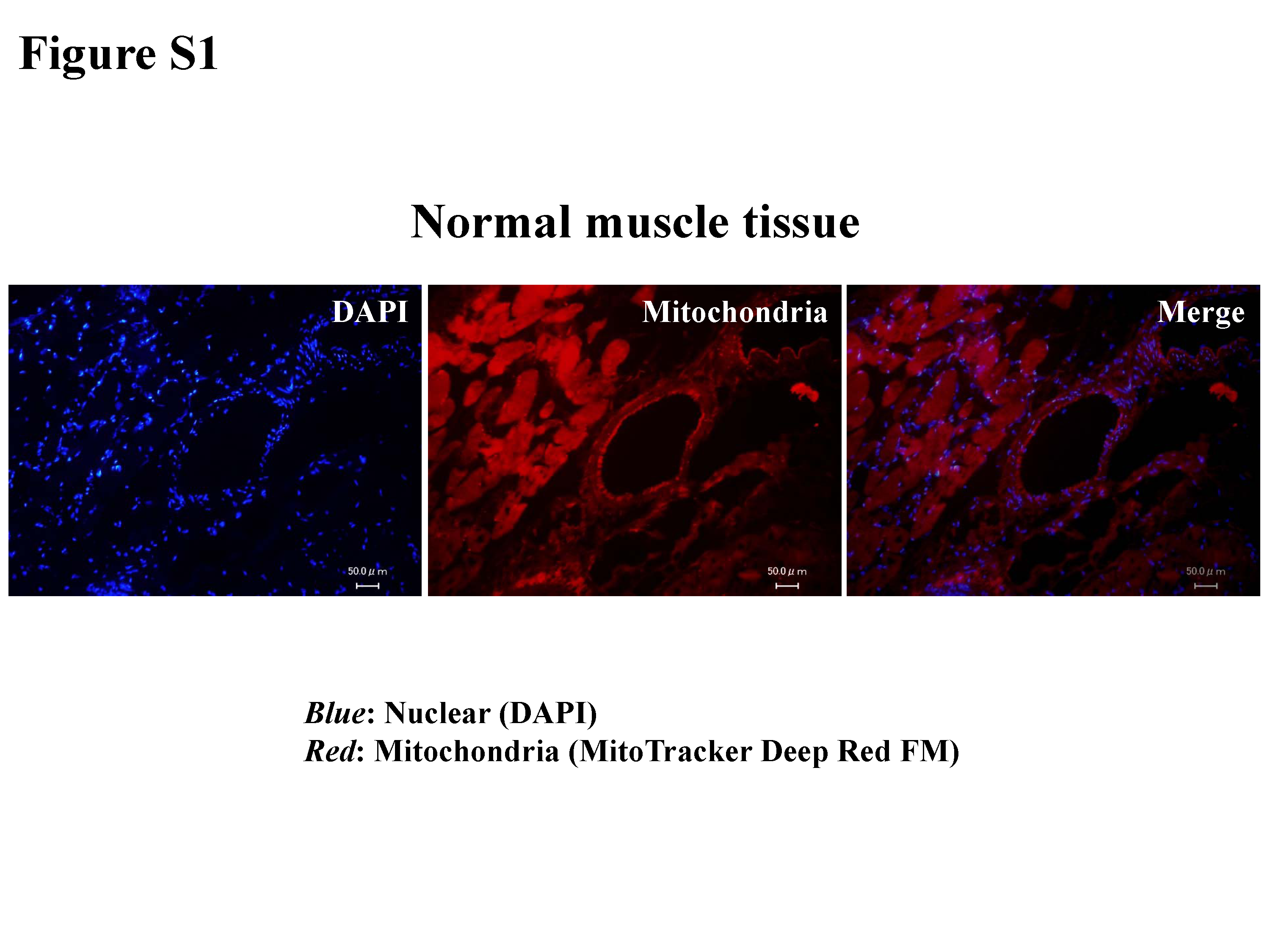

Supplement: Figure S1 — Immunofluorescence staining were performed in normal muscle tissues of mice as the control images of staining using the MitoTracker Deep Red FM (Invitrogen). The nucleus was stained with DAPI. The images were obtained using a BZ-8000 confocal microscope (Keyence). (TIFF) [file pone.0049189.s001.tiff]

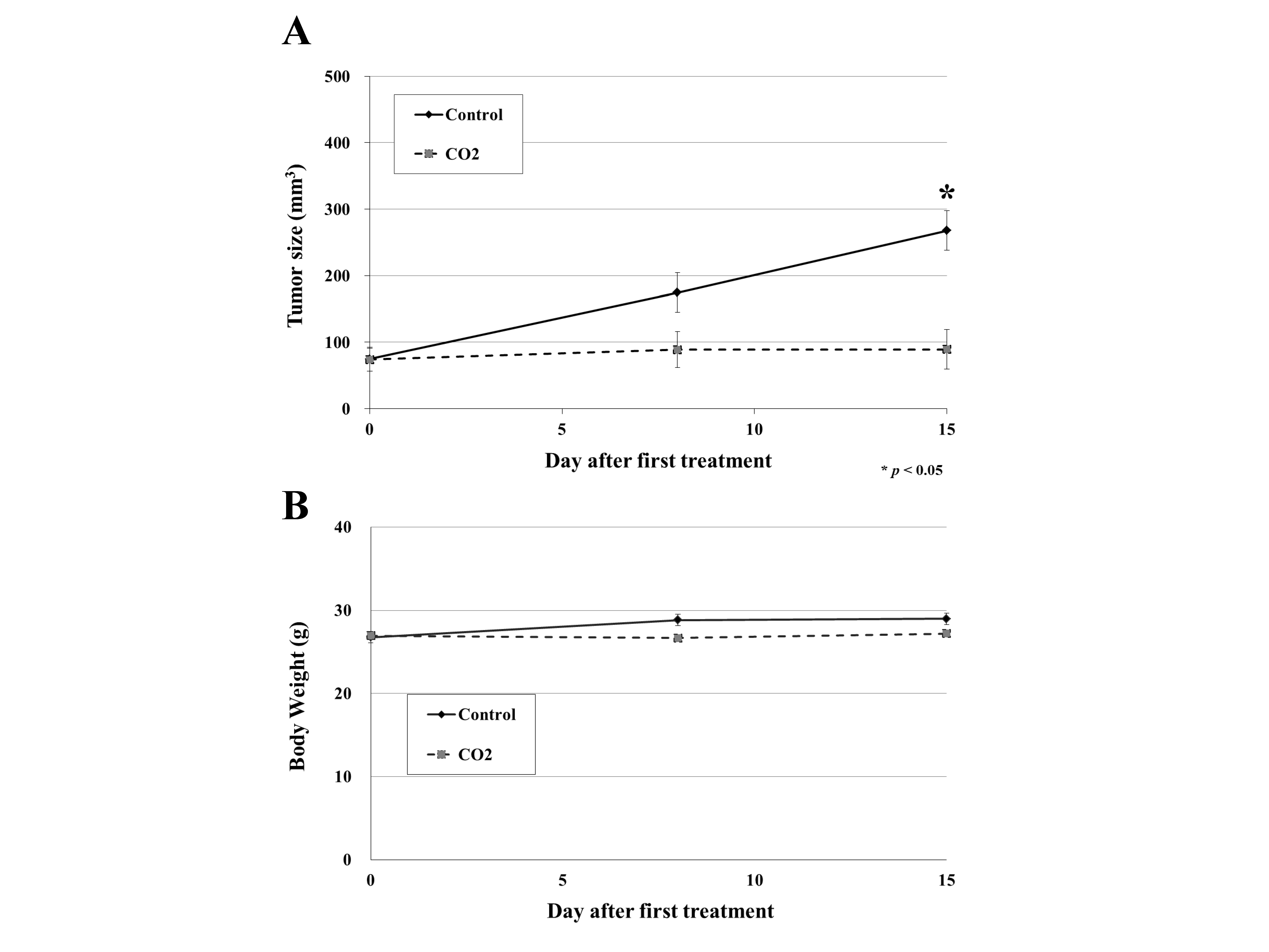

Supplement: Figure S2 — Effect of our transcutaneous CO2 treatment on the in vivo tumor growth of human breast cancer cell line, MDA-MB-231. Tumor model mice were created by subcutaneous implantation of the cells (1.5×10^6^ cells in 500 µl PBS). Mice were randomly divided into CO2 group (n = 5) or control group (n = 5), and treatment was performed twice weekly for 15 days. Tumor volume (A) and body weight (B) in mice were monitored until the end of the treatment. (A) At the end of the treatment, we observed a significant decrease in tumor volume in CO2 group compared with the control group (*p<0.05). (B) No significant difference in body weight was observed between CO2 treated and control groups. (TIFF) [file pone.0049189.s002.tiff]

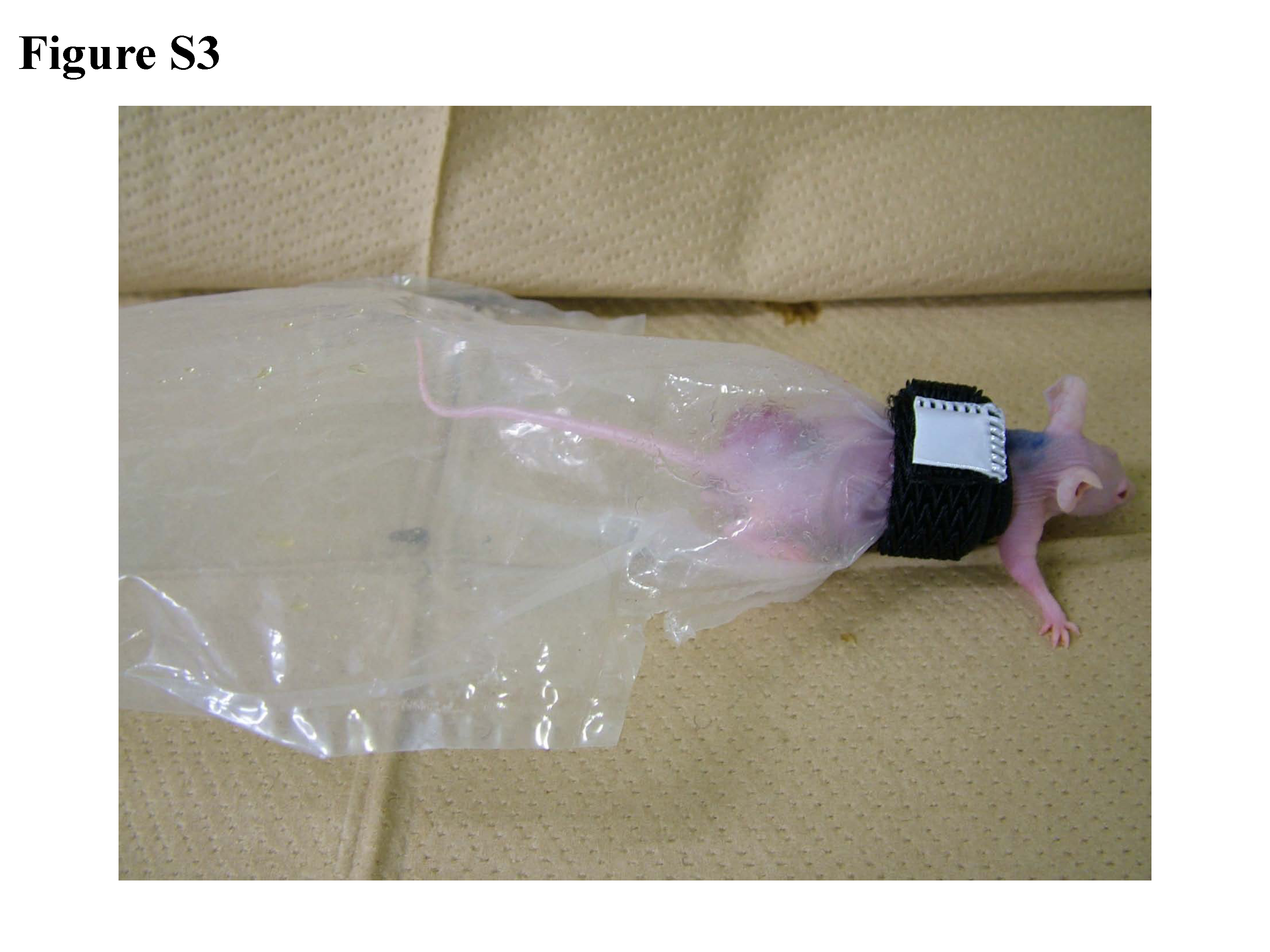

Supplement: Figure S3 — Transcutaneous application of CO2 for a model mouse of human MFH. (TIFF) [file pone.0049189.s003.tiff]
